# Increased Soluble CD137 Levels and CD4+ T‐Cell‐Associated Expression of CD137 in Acute Atherothrombotic Stroke

**DOI:** 10.1111/cts.12553

**Published:** 2018-04-26

**Authors:** Yang He, Dong‐Hui Ao, Xiao‐Qing Li, Shan‐Shan Zhong, Rong A, Yang‐Yang Wang, Ya‐Juan Xiang, Bao‐Lei Xu, Ting‐Ting Yang, Xu‐Guang Gao, Guang‐Zhi Liu

**Affiliations:** ^1^ Department of Neurology Peking University People's Hospital Beijing China; ^2^ Department of Neurology Beijing Anzhen Hospital Capital Medical University Beijing China

## Abstract

As a proinflammatory cytokine, CD137 (4‐1BB, TNFRSF9) is present in membrane‐bound and soluble forms. Increased expression of CD137 was recently found in T cells in human atherosclerotic plaques. However, the exact role of CD137 in ischemic stroke is not clear. In this study we analyzed the protein levels of soluble CD137 (sCD137) and the expression of CD137 on CD4+ T cells in the peripheral blood of patients with acute atherothrombotic stroke by using the cytometry beads array (CBA) and flow cytometry. Within 24 hours of onset, the stroke patients showed elevated levels of sCD137 (2.7 pg/ml) and CD137 expression on CD4+ T cells (4.9 ± 3.2%) compared with normal controls (1.1 pg/ml, P < 0.01; 1.3 ± 1.0%, P < 0.01). Alterations in CD137 expression may enhance ischemia‐induced inflammatory responses via bidirectional signaling and, consequently, aggravate brain injury in early stages of this disorder.

**Study Highlights**

**WHAT IS THE CURRENT KNOWLEDGE ON THE TOPIC?**

✓ The CD137/CD137L pathway plays proatherogenic or pathogenic roles in the process of atherosclerosis‐related disorders. The role of CD137 in atherothrombotic stroke remains unknown.

**WHAT QUESTION DID THIS STUDY ADDRESS?**

✓ We asked whether CD137 is aberrantly produced in acute atherothrombotic stroke and we discussed its clinical relevance.

**WHAT DOES THIS STUDY ADD TO OUR KNOWLEDGE?**

✓ A remarkable increase in the expression of CD4+ T cells and plasma levels of CD137 were found in patients with acute ischemic atherosclerotic stroke. Moreover, the elevated CD137 surface expression or sCD137 protein levels were significantly correlated with NIHSS and infarct volume, respectively.

**HOW MIGHT THIS CHANGE CLINICAL PHARMACOLOGY OR TRANSLATIONAL SCIENCE?**

✓The alteration in CD137 expression may enhance ischemia‐induced inflammatory responses via bidirectional signaling and, consequently, aggravate brain injury in the early stages of this disease. Our findings are considerably useful for identifying potential biomarkers in atherothrombotic stroke and, more important, paving the way for exploring a new therapeutic strategy against this human disorder via intervention of the CD137/CD137L pathway.

Atherosclerosis is a chronic inflammatory disease that is initiated by cholesterol deposition, particularly oxidized low‐density lipoprotein (oxLDL), in the intimal layer of arteries.^1^, ^2^ Growing evidence indicates that both innate and adaptive immune responses participate in the initiation and progression of atherosclerosis, with the involvement of immune cells such as CD4+ T cells, monocytes, or macrophages in the arteries; systemic elevation of proinflammatory cytokines; matrix metalloproteinases (MMP) and tissue factor (TF).^3^, ^4^ Advanced atherosclerosis can contribute to the production of arterial thrombi, which may lead to heart attack and ischemic stroke.^5^

To date, various studies have provided strong evidence that tumor necrosis factor (TNF) superfamilies (TNFSF), as proinflammatory cytokines, are implicated in the pathogenesis of atherosclerosis.^3^, ^6^ TNF‐α and CD40 ligand (CD40L) have been found to play important roles in atherogenesis by provoking immune responses such as the release of proinflammatory cytokines, MMP activation, and oxLDL‐induced TF expression.^7^, ^8^, ^9^ In addition, increased expression of another TNFSF member, CD137 (4‐1BB, TNFRSF9), has been reported on T cells in human atherosclerotic plaques and the endothelium, and it is able to induce adhesion molecule expression in the endothelium and reduce smooth muscle cell proliferation upon activation by CD137 ligands (CD137L, 4‐1BBL, TNFSF9).^10^ In an experimental study of CD137‐deficient apolipoprotein E‐knockout mice (*ApoE^−^*/*^−^CD137^−^*/^−^) and low‐density lipoprotein (LDL)‐receptor–knockout mice (*Ldlr^−^*/*^−^CD137^−^*/^−^), the lack of CD137 produced fewer atherosclerotic plaques in these two models, whereas stimulation of CD137L signaling by soluble CD137 activated monocytes/macrophages and augmented the secretion of proinflammatory cytokines in atherosclerotic plagues.^11^, ^12^ Furthermore, two distinct studies reported that patients with acute coronary syndromes demonstrated significantly higher CD137 expression in monocytes and greater levels of serum soluble CD137 (sCD137) than control groups.^13^, ^14^ Taken together, all these findings strongly support the pro‐atherogenic or pathogenic roles of the CD137/CD137L pathway in the process of atherosclerosis‐related coronary heart disease. To clarify the role of this pathway in cerebrovascular disease, we conducted a pilot study and analyzed CD137L levels in patients with acute atherothrombotic stroke. We found that stroke patients had elevated plasma soluble CD137L levels and increased CD137L expression in monocytes, suggesting a dysregulation of CD137/CD137L signaling in the early stage of this disorder.^15^ To verify these findings, it is necessary to conduct further studies on CD137 expression and explore its roles in the pathogenesis of ischemic stroke. In summary, we analyzed the surface expression of CD137 and soluble CD137 (sCD137) proteins in peripheral blood of patients with acute ischemic atherosclerotic stroke to determine whether this molecule is aberrantly produced in this disorder, as well as its clinical relevance.

## METHODS

### Patients and controls

A total of 27 patients with acute ischemic stroke (17 males and 10 females; mean age 65.6 ± 11.8 years) were included in this study. All of them had stroke symptoms present on awakening (wakeup stroke) between 5–7 am. These patients belonged to the large‐artery atherosclerosis (LAA) subtype, according to the Acute Stroke Treatment (TOAST) classification.^16^ All patients were diagnosed within 24 hours of the onset of symptoms in terms of medical history, clinical examination, and cranial magnetic resonance image (MRI) and magnetic resonance angiography (MRA) scans. Carotid duplex sonography was carried out in all patients, particularly for measuring total plaque area (TPA), as described elsewhere.^17^, ^18^ The National Institutes of Health Stroke Scale (NIHSS) was used to assess neurological deficits while taking blood samples. Infarct volume was calculated by summing the number of outlined voxels and multiplying by the slice thickness on each slice where the lesion was visible on axial diffusion‐weighted imaging (DWI). Patients with cardiogenic stroke were completely excluded from this study. In addition, five of these stroke patients on atorvastatin (80 mg q.d.) treatment were selected for serial study, and did not receive any therapy prior to the investigation.

A total of 19 patients with asymptomatic carotid stenosis (ACS) (>50%) (13 males and 6 females; mean age 69.0 ± 9.8 years) were enrolled as atherosclerosis controls on carotid duplex sonography. These patients had no history of ischemic stroke on the basis of clinical and cranial MRI evaluation. A total of 20 healthy subjects were recruited as normal controls (NC) (9 males and 11 females; mean age 71.4 ± 10.6 years); they had no evidence of large‐artery atherosclerosis, history of stroke, history of heart attack, or vascular risk factors.

None of the participating individuals had i) an infectious disease; ii) autoimmune, renal, hepatic, or cancerous disorders; iii) previously received statin therapy. Informed consent for all participants was completed and the study was approved by the Ethics Committee of Peking University People's Hospital.

### Blood sampling

The heparinized venous blood specimens were obtained from 09:00 am to 12:00 pm. Peripheral blood mononuclear cells (PBMC) were isolated from the blood samples by standard density gradient centrifugation. After a brief centrifugation, plasma was collected and frozen as separate aliquots at –70°C; plasma was subsequently thawed immediately prior to the assay.

### Flow cytometry

Freshly isolated PBMC were characterized for CD137 expression by three‐color direct immunofluorescence via flow cytometry using a FACScan device (Becton Dickinson, San Jose, CA). The following antibodies were added to 2 × 10^5^ cells in 200 μl cell suspension: i) fluorescein isothiocyanate (FITC)‐labeled anti‐IgG1 (Becton Dickinson), allophycocyanin (APC)‐labeled anti‐IgG1, peridinin chlorophylla protein (PerCP)‐labeled anti‐CD4 (R&D Systems, Minneapolis, MN); ii) FITC‐labeled anti‐CD28 (Becton Dickinson), APC‐labeled anti‐CD137, PerCP‐labeled anti‐CD4 (R&D Systems). After incubation at 4°C for 30 minutes, cells were washed twice with staining buffer and analyzed on the FACScan using CellQuest software.

### Cytometry beads array (CBA)

Plasma sCD137 levels were determined with a commercial AimPlex Analyte Kit according to the supplier's instructions (Aimplex, Beijing, China), with a detection sensitivity of 1 pg/ml. All assays were carried out on FACScan in duplicate. To evaluate the effect of freezing and thawing on plasma samples, we stored the plasma samples at −70°C for 24 hours and then allowed them to thaw at room temperature for 30 minutes. This preliminary experiment demonstrated no significant difference of sCD137 levels between freshly isolated plasma and plasma from one individual after one freeze–thaw cycle (data not shown).

### Statistics

The data are presented as mean ± standard deviation (CD137 surface expression and frequency of CD4+CD28– T cells) or median with range (infarct volume, sCD137). The normal distributed data were processed using one‐way analysis of variance (ANOVA) and Student–Newman–Keul's *post‐hoc* test and Pearson's correlation test. Nonnormal distributed data were analyzed with Kruskal–Wallis ANOVA and Spearman's correlation test. *P* ≤ 0.05 was considered statistically significant and *P* ≤ 0.01 was considered highly statistically significant. To estimate the sample size, a pilot study was conducted for measuring CD137 expression in a few patients with stroke (*n* = 10), asymptomatic carotid stenosis (*n* = 10), and normal controls (*n* = 10). The mean ± standard deviation of plasma sCD137 levels of these three groups was 3.45 ± 2.08, 2.11 ± 0.67, and 1.23 ± 0.53 pg/ml, respectively. With α = 0.05, two‐tailed and a power of 90%, we needed nine patients per group. Considering a compliance rate of 80%, we asked at least 34 patients to participate in this study.

## RESULTS

### Basic characteristics

Patient's clinical and laboratory data collected at the time of blood sampling are presented in **Table** 1; stroke patients had significantly higher percentages of smoking habits and blood leukocyte counts than NC.

**Table 1:** Baseline characteristics of patients and normal controls

	Ischemic stroke (*n =* 27)	Asymptomatic carotid stenosis (*n =* 19)	Normal controls (*n =* 20)	*P* value
Sex (male/female)	17/10	13/6	9/11	—
Age (years)	65.6±11.8	69.0±9.8	71.4±10.6	0.1933
Hypertension (%)	48.2	84.2	—	—
Diabetes mellitus (%)	37.0	52.6	—	—
Smoking (%)	33.3	57.9	20.0	0.0444
Hypercholesterolemia (%)	88.9	68.4	70.0	0.1717
Peripheral artery disease (%)	22.2	21.1	—	—
Leukocyte (×10^3^/μl)	7.4±1.7	6.6±1.8	6.1±1.7	0.0296
Lymphocyte (×10^3^/μl)	1.9±0.7	1.7±0.6	1.8±0.7	0.6652
Total plaque area (mm^2^)	56.8±34.6	110.0±64.2	—	—
NIHSS	6.8±3.3	—	—	—
*Infarct volume (cm^3^)	6.3 (3.25–55.44)	—	—	—

Data are mean ± SD; NIHSS: National Institutes of Health Stroke Scale.

Data of infarct volume are median with range.

### Cell surface expression of CD137

**Figure 1:**
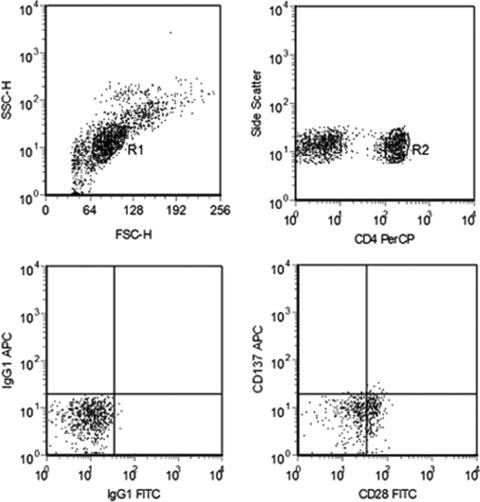
Flow cytometry. Region 1 (R1) is selected to set a mononuclear cell gate according to forward light scatter (FSC) and side light scatter (SSC) properties. Region 2 (R2) is used to set a CD4+ T‐cell gate for further CD137 analysis. Control staining with isotype control antibodies was used as a control to define the gate.

**Figure 2:**
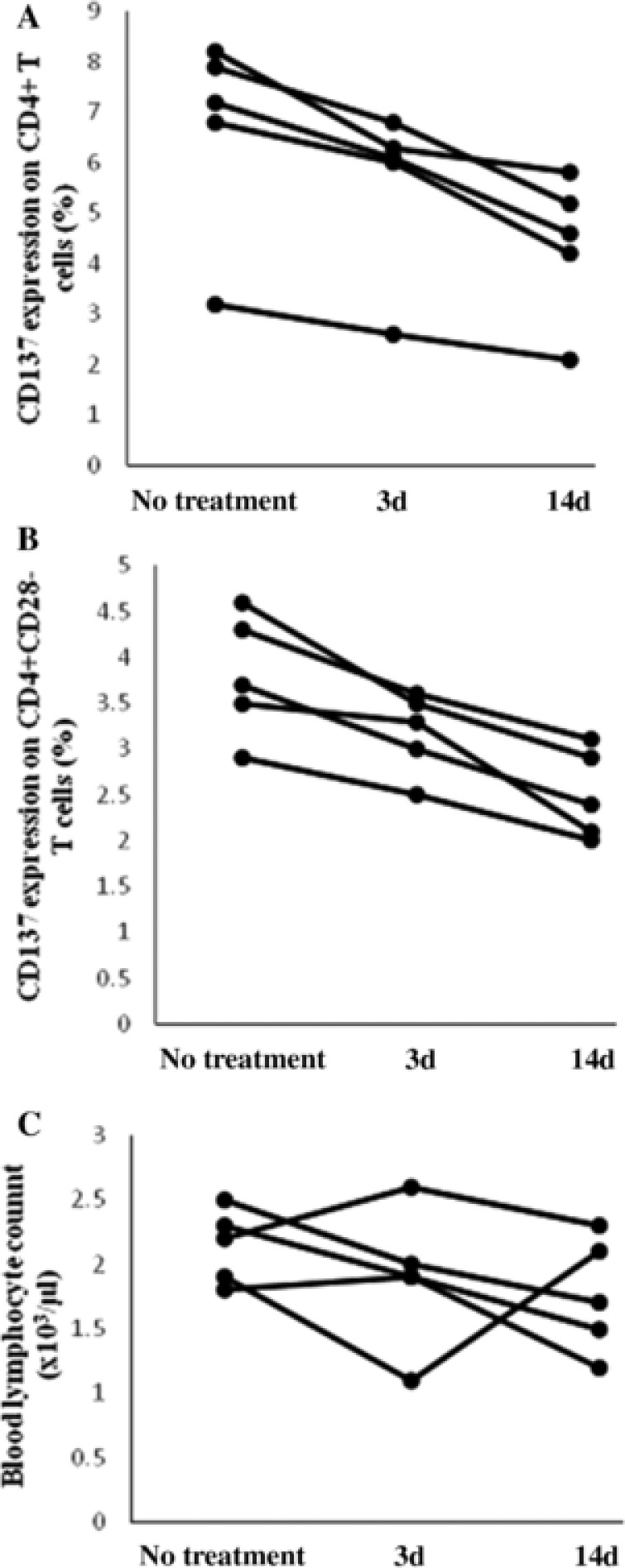
Changes of CD137 expression on CD4+ and CD4+CD28– T cells in stroke patients after treatment. Changes of CD137 expression on CD4+ and CD4+CD28– T cells (a,b), as well as changes in the blood lymphocyte count (c) were observed in five patients with acute ischemic atherosclerotic stroke before treatment and after 3 and 14 days of treatment with statin; 3 d = 3 days; 14 d = 14 days.

Within 24 hours of onset, the stroke patients showed an increased frequency of CD4+CD28– T cells (14.1 ± 4.4%) as well as elevated expression of CD137 (**Figure** 1) on CD4+ (4.9 ± 3.2%) and CD4+CD28– T cells (2.6 ± 2.1%) compared with the ACS and NC groups (CD4+CD28–: 9.0 ± 4.6% and 8.1 ± 3.3%, *P =* 0.0004 and *P <* 0.0001; CD137 expression on CD4+ T cells: 2.2 ± 1.7% and 1.3 ± 1.0%, *P =* 0.0018 and *P <* 0.0001; CD137 expression on CD4+CD28– T cells: 0.7 ± 0.7% and 0.5 ± 0.3%, *P =* 0.0004 and *P <* 0.0001), but no significant differences were found between the ACS group and the NC group (**Table** 2). Moreover, five stroke patients appeared to have increased baseline CD137 expression (CD4+: 6.7 ± 2.0%; CD4+CD28–: 3.8 ± 0.6%) compared with the rest of the population, but all of these patients demonstrated a continuous decrease in CD137 expression on CD4+ and CD4+CD28– T cells at 3 and 14 days posttreatment, while the lymphocytes presented irregular variations (**Figure** 2).

**Table 2:** CD4+ and CD4+CD28– T cells spontaneously expressing CD137 in peripheral blood of patients with acute ischemic atherosclerotic stroke, asymptomatic carotid stenosis (ACS), and normal controls (NC)

		CD137 (%)	
		
	CD4+CD28– (%)	CD4+	CD4+CD28–
Stroke (*n =* 27)	14.1±4.4^a^	4.9±3.2^a^	2.6±2.1^a^
ACS (*n =* 19)	9.0±4.6	2.2±1.7	0.7±0.7
NC (*n =* 20)	8.1±3.3	1.3±1.0	0.5±0.3
*P* value (ANOVA)	<0.0001	<0.0001	<0.0001

a *P* < 0.01 for *post‐hoc* comparison with either of control groups.

### Plasma sCD137 levels

**Figure 3:**
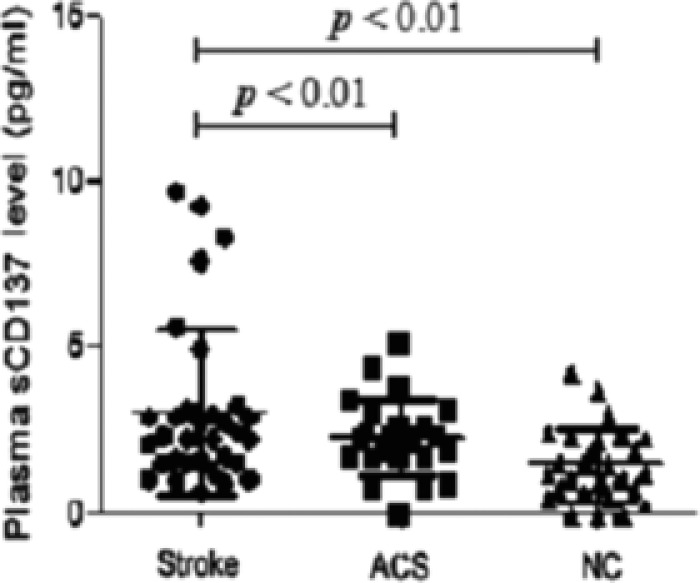
Comparison of plasma soluble CD137 (sCD137) levels between stroke patients and control groups. Comparison of plasma sCD137 levels between patients with acute ischemic atherosclerotic stroke, asymptomatic carotid stenosis (ACS) and normal controls (NC) using a cytometry beads array (CBA). Horizontal lines indicate median values with range, numerals on top are *P*‐values.

**Figure 4:**
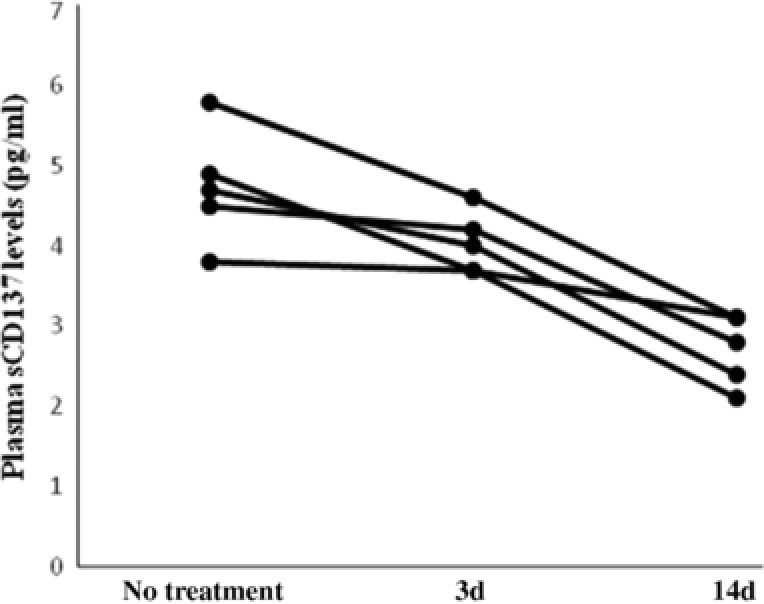
Changes of plasma soluble CD137 (sCD137) levels in stroke patients after treatment. Changes of plasma sCD137 levels were observed in five patients with acute ischemic atherosclerotic stroke before treatment and after 3 and 14 days of treatment with statin; 3 d = 3 days; 14 d = 14 days.

Within 24 hours of symptom onset, the plasma sCD137 levels were significantly higher in the stroke patients (2.7 pg/ml (range: 1.1–9.7)) than the NC group (1.1 pg/ml (range: 0.3–3.0), *P <* 0.0001), and there was also a significant difference between the ACS group and the NC group (2.1 pg/ml (range: 0.8–3.9) vs 1.1 pg/ml (range: 0.3–3.0), *P =* 0.0027) (**Figure** 3). In addition, five stroke patients receiving statin therapy demonstrated a decreasing trend of plasma sCD137 levels at 3 and 14 days posttreatment (**Figure** 4), as these patients appeared to have higher baseline sCD137 levels (3.3 pg/ml (range: 2.9–5.6) than the rest of the population.

### Correlation analysis

The results showed a correlation of the surface expression of CD137 on CD4+ T cells and levels of plasma sCD137 with NIHSS (*r =* 0.7167, *P <* 0.0001 and *r =* 0.7119, *P =* 0.0137) and infarct volume (*r =* 0.6207, *P =* 0.0006 and *r =* 0.7072, *P <* 0.0001) in stroke patients. A positive correlation was shown between the sCD137 levels and CD137 expression on CD4+ T cells (*r =* 0.7694, *P <* 0.0001) as well as frequency of CD4+CD28– T cells (*r =* 0.3835, *P =* 0.0483). In addition, there was no correlation of sCD137 levels or CD137 surface expression on CD4+ or CD4+CD28– T cells with the TPA of stroke patients (data not shown).

## DISCUSSION

CD137 is expressed by various immune cells, such as activated CD4+ and CD8+ T lymphocytes, natural killer (NK) cells, CD4+CD25+ regulatory T cells (Treg), monocytes, neutrophils, mast cells, eosinophils, and dendritic cells^9^, ^10^, ^11^, ^12^, ^13^, ^14^, ^15^, ^16^, ^17^, ^18^, ^19^, ^20^, ^21^ as well as nonimmune cells such as endothelial cells and vascular smooth muscle cells.^10^ The ligand of CD137 (CD137L) is primarily expressed on antigen‐presenting cells (dendritic cells, monocytes/macrophages, and B cells), human primary T cells^22^ and cardiac myocytes in myocarditis and aortic tissue in arteritis.^23^, ^24^ In addition to mediating costimulation on T‐cell activation and survival, the engagement of CD137L with soluble CD137 can also elicit reverse signaling and, in turn, stimulate the activation, migratory capabilities, and survival of monocytes expressing CD137L.^25^, ^26^ In the present study, patients with acute ischemic atherosclerotic stroke showed an increase in CD137 expression on CD4+ T cells and plasma levels of sCD137 compared with control groups, indicating that the CD137‐mediated immune response was involved in the disease pathogenesis. Together with our previous study revealing higher CD137L expression on CD14+ monocytes,^15^ these findings support the view that dysregulation of CD137L/CD137 signaling may occur in the early stage of acute ischemic atherosclerotic stroke.

CD4+CD28– (CD28null) T cells, as a subset of CD4+ T cells that lack the costimulatory receptor CD28, have unique immune functions in terms of their secretion of proinflammatory cytokines, namely, interferon (IFN)‐γ and TNF‐α.^27^, ^28^ Moreover, CD28– T cells were shown to produce the cytotoxic molecules perforin and granzyme B, which resulted in cytolysis of the endothelium *in vitro*.^29^, ^30^ CD4+CD28– T cells have been found in patients with cerebrovascular and cardiovascular diseases such as acute coronary syndrome^27^, ^31^ and stroke.^32^, ^33^, ^34^ Of note, in these patients this proinflammatory cytokine production is upregulated by the costimulatory receptors OX40 and CD137 on circulating CD4+CD28– T cells,^25^ which were also found to be located in atherosclerotic plaques preferentially accumulating in unstable lesions.^35^ Consistent with these previous studies, our study showed that, compared with control groups, patients with acute atherothrombotic stroke had a marked increase of CD4+CD28– T cells, validating a derangement of this cell population occurring in the inflammatory process of this disorder. Furthermore, elevated CD137 expression on CD4+CD28– T cells in stroke patients may be responsible for this cellular dysregulation. Interestingly, no differences in the frequency of CD4+CD28– T cells or CD137 expression in CD4+CD28– T cells were found between the ACS and NC groups, since the former category of patients was previously reported to exhibit CD4+CD28– T cells located in lesions with plaques rupture.^35^ However, more evidence is needed to address these issues.

Restricted knowledge is currently provided concerning roles of the CD137L/CD137 pathway in atherosclerosis, largely because of the complexity of bidirectional signaling through CD137 and its ligand. In the CD137‐deficient atherosclerotic vessels of ApoE^−^/^−^CD137^−^/^−^ mice, crosslinking CD137L activation with soluble CD137 protein *ex vivo* activated CD137L signaling and led to the release of proinflammatory cytokines, such as TNF‐α and monocyte chemotactic protein‐1 (MCP‐1).^11^ In ACS patients, blocking the CD137 pathway *in vitro* remarkably reduced the production of TNF‐α and IFN‐γ from circulating CD4+CD28– T cells, which were reported to induce rupture of atherosclerotic plaques by direct cytolysis of arterial smooth muscle and endothelial cells.^27^, ^30^ In our serial study, despite an irregular change of lymphocyte counts in the stroke patients, CD137 expression in the CD4+ and CD4+CD28– T cells displayed a decreasing trend at 3 and 14 days posttreatment with statin, a well‐known cholesterol‐lowering drug with multiple antiinflammatory properties.^36^ Together with a stroke study revealing markedly decreased blood TNF‐α levels after a short‐time use of statin,^37^ our findings suggest a positive regulatory role of membrane‐bound CD137 in the disease pathogenesis, probably via cell‐to‐cell interaction with CD137L for delivery of costimulatory signals that stimulate CD4+ T cells immunoactivities, such as secretion of TNF‐α and IFN‐γ, and as a result, aggravate ischemic injury in the brain. The positive correlation between CD137 expression on CD4+ T cells and infarct volume or disease severity in the present study further supports this view.

With regard to the elevated plasma sCD137 levels, we initially assumed that the increased sCD137 production may predominately result from the upregulated CD137 expression on CD4+ T cells of stroke patients. As expected, our study showed a positive correlation between the sCD137 levels and CD137 surface expression on CD4+ T cells. This is noteworthy since, under physiological conditions, sCD137 is generated by proteolytic cleavage from the cell surface of activated lymphocytes.^38^, ^39^ An upregulated specific MMP, i.e., gelatinase B and MMP‐9, which could cause the cleavage of CD137 from the cell surface during the acute stage of stroke,^40^, ^41^ may contribute to the increased release of sCD137. Our study demonstrated that plasma sCD137 levels tended to decrease following treatment, in parallel with the decreasing serum MMP‐9 levels after statin therapy.^42^, ^43^ Unlike membrane‐bound CD137 that delivers a potent stimulatory signal to activate T cells, sCD137 may enhance immune responses by reverse signaling, as shown in an *ex vivo* study by Jeon *et al*., who showed that sCD137‐treated macrophages expressing CD137L can produce MCP‐1 and TNF‐α.^11^ Taking our additional results into consideration, i.e., the positive correlation of sCD137 levels with brain infarct volume and disease severity, the shedding of CD137 from lymphocytes, such as activated CD4+ T cells, may function as a positive feedback mechanism to further promote brain ischemia‐related inflammation that has been triggered by the local cell–cell CD137/CD137L interaction in the acute stage of stroke. Indeed, as shown in **Figure** 3, six stroke patients showing seemingly elevated plasma sCD137 levels (8.0 pg/ml (range: 4.9–9.7)) appeared to have higher NIHSS (10.7 ± 1.8) and larger infarct volume (21.6 cm^3^ (range: 11.7–55.4)) compared with the rest of the population. Conversely, the immunological abnormality of CD137 could be a general phenomenon in chronic inflammation, since elevated sCD137 levels have been also described in other inflammatory disorders.^13^, ^44^, ^45^

In this study we found remarkably increased surface expression and plasma levels of CD137 in patients with acute ischemic atherosclerotic stroke. This validates our view that dysregulation of CD137L/CD137 signaling occurs in the early stage of this disorder. More strikingly, both elevated sCD137 protein and CD137 surface expression levels were demonstrated to—at least partially—reflect the disease severity and brain injury during the acute stage. Nevertheless, more studies (e.g., prospective cohort designs) are needed to ascertain whether these two parameters could be utilized as predictive biomarkers for atherothrombotic stroke, as well as their sensitivity and specificity, since there are other diseases, such as oncological malignancies, which also present elevated CD137 levels, thus indicating that these markers could be nonspecific for stroke outcome measures. In addition, other issues should also be addressed, including prognostic vs. nonprognostic characteristics of CD137 as a biomarker to predict an acute episode in patients diagnosed with asymptomatic carotid stenosis. Regarding the roles of CD137 in disease pathogenesis, our findings strongly suggest that the abnormal membrane‐bound expression of CD137 may play a positive regulatory role in the process of ischemic injury via delivery of costimulatory signals for activation of CD4+ T cells; furthermore, enhanced plasma levels of sCD137 could stimulate a positive feedback effect that further promotes brain ischemia‐related inflammatory responses by reverse signaling. Therefore, our novel discoveries in CD137 expression in atherothrombotic stroke certainly pave the way for identifying potential biomarkers in atherothrombotic stroke and, more importantly, exploration of a new therapeutic strategy against this human disorder via intervention of the CD137/CD137L pathway.

## Conflict of Interest

The authors declare no competing interests for this work.

## Supporting information

DATASET_CD137_STROKE
